# Effect of Clarithromycin, a Strong CYP3A and P-glycoprotein Inhibitor, on the Pharmacokinetics of Edoxaban in Healthy Volunteers and the Evaluation of the Drug Interaction with Other Oral Factor Xa Inhibitors by a Microdose Cocktail Approach

**DOI:** 10.1007/s10557-023-07443-2

**Published:** 2023-03-04

**Authors:** Alexander Lenard, Simon A. Hermann, Felicitas Stoll, Juergen Burhenne, Kathrin I. Foerster, Gerd Mikus, Andreas D. Meid, Walter E. Haefeli, Antje Blank

**Affiliations:** 1 Department of Clinical Pharmacology and Pharmacoepidemiology, Heidelberg University Hospital, University of Heidelberg, Im Neuenheimer Feld 410, 69120 Heidelberg, Germany; 2 Partner Site Heidelberg, German Center for Infection Research, Heidelberg, Germany

**Keywords:** Factor Xa inhibitors, Clarithromycin, Drug-drug interaction, Healthy volunteers, Microdose

## Abstract

**Purpose:**

We assessed the differential effect of clarithromycin, a strong inhibitor of cytochrome P450 (CYP) 3A4 and P-glycoprotein, on the pharmacokinetics of a regular dose of edoxaban and on a microdose cocktail of factor Xa inhibitors (FXaI). Concurrently, CYP3A activity was determined with a midazolam microdose.

**Methods:**

In an open-label fixed-sequence trial in 12 healthy volunteers, the pharmacokinetics of a microdosed FXaI cocktail (μ-FXaI; 25 μg apixaban, 50 μg edoxaban, and 25 μg rivaroxaban) and of 60 mg edoxaban before and during clarithromycin (2 x 500 mg/d) dosed to steady-state was evaluated. Plasma concentrations of study drugs were quantified using validated ultra-performance liquid chromatography–tandem mass spectrometry methods.

**Results:**

Therapeutic clarithromycin doses increased the exposure of a therapeutic 60 mg dose of edoxaban with a geometric mean ratio (GMR) of the area under the plasma concentration-time curve (AUC) of 1.53 (90 % CI: 1.37–1.70; *p* < 0.0001). Clarithromycin also increased the GMR (90% CI) of the exposure of microdosed FXaI apixaban to 1.38 (1.26–1.51), edoxaban to 2.03 (1.84–2.24), and rivaroxaban to 1.44 (1.27–1.63). AUC changes observed for the therapeutic edoxaban dose were significantly smaller than those observed with the microdose (*p* < 0.001).

**Conclusion:**

Clarithromycin increases FXaI exposure. However, the magnitude of this drug interaction is not expected to be clinically relevant. The edoxaban microdose overestimates the extent of the drug interaction with the therapeutic dose, whereas AUC ratios for apixaban and rivaroxaban were comparable to the interaction with therapeutic doses as reported in the literature.

**Trial Registration:**

EudraCT Number: 2018-002490-22

**Supplementary Information:**

The online version contains supplementary material available at 10.1007/s10557-023-07443-2.

## Introduction

Since their approval, direct-acting oral factor Xa inhibitors (FXaI) have rapidly rising prescription rates because of at least similar effectiveness to vitamin K antagonists, less variable dosing regimens, fewer monitoring requirements, and a favorable safety profile [1–3]. Their effects are immediate and concentration-dependent, indicating that changes in dose or clearance will immediately translate into exposure and effect changes. Depending on the indication, FXaI maintenance doses differ, and, as shown for rivaroxaban, rather, small dose steps of 50 % or 25 % of the maximum 20 mg dose can be of importance, indicating that appropriate FXaI dose selection is critical [4–6].

Different pathways of transport, metabolism, and elimination are involved in the pharmacokinetics of currently available FXaI, and their relative contribution to clearance varies among different FXaI [5]. Accordingly, impairment of these pathways by drug-drug interactions (DDI) caused by co-administered drugs can result in variable exposure changes that are difficult to predict. Therefore, pharmacokinetic DDI are not a class phenomenon and need to be tested or modelled for each compound separately.

The currently marketed FXaI are substrates of drug transporters such as P-glycoprotein (P-gp, ABCB1), are metabolized by various phase-I (cytochrome P450 (CYP) 3A, CYP2J2, and carboxylesterase 1) and phase-II enzymes (e.g., UDP-glucuronosyltransferases), and are partly eliminated renally. But not all pathways are equally relevant for each compound [5, 7–9]; as an example, edoxaban is only minimally metabolized by CYP, whereas the CYP contribution to overall apixaban and rivaroxaban clearance is 21 and 32 %, respectively. In addition, applying results of DDI studies with FXaI to individual patient settings is complicated by the large interindividual variability in the pharmacokinetics of FXaI [10–12].

As different as the individual metabolic pathways of FXaI are, so is the extent of interaction with a particular perpetrator drug [5]. It is therefore important to evaluate pharmacokinetic drug interactions for each individual victim drug. An attractive way to study the interaction profile of an entire drug class is to administer these drugs simultaneously (cocktail) in microdoses, minimizing inter-day and inter-subject variability and increasing statistical power [13]. This is particularly important for narrow therapeutic index compounds such as FXaI to minimize bleeding risks. By administering all three FXaI as a microdose cocktail, it is therefore possible to reduce intraindividual and interindividual variability with negligible effects on coagulation, while simplifying study conduct and minimizing time, risk, and cost [14].

Clarithromycin is a known CYP3A inhibitor, which increased apixaban concentrations by 60 % [15], and rivaroxaban by 50–100 % [11, 16] in previous trials and which was associated with bleeding events [17, 18]. In a clinical trial in healthy volunteers, we assessed the yet unknown effect of the macrolide antibiotic clarithromycin on the pharmacokinetics of a therapeutic dose of edoxaban. In addition, we evaluated the effect of clarithromycin on the pharmacokinetics of apixaban, edoxaban, and rivaroxaban administered as a microdosed FXaI cocktail to further evaluate the utility of such a cocktail for assessing pharmacokinetic DDI with these FXaI.

## Materials and Methods

### Ethics Approval

The study protocol was approved by the competent authority (BfArM, Bonn, Germany, Vorlagen Nr. 4043377) and received a positive vote of the responsible Ethics Committee of the Medical Faculty of Heidelberg University, Germany (AFmo-144/2019), and the trial was registered in the EudraCT database (EudraCT 2018-002490-22). This phase I, investigator-initiated, monocenter trial was conducted in accordance with the Declaration of Helsinki, the principles of Good Clinical Practice, and all pertinent legal requirements at the DIN EN ISO 9001-certified early clinical trial unit KliPS of the Department of Clinical Pharmacology and Pharmacoepidemiology, Heidelberg University Hospital.

### Trial Population and Design

Healthy volunteers between 18 and 65 years were eligible after full information and after having given their written informed consent. Inclusion and exclusion criteria ensured the enrolment of volunteers in good health without any relevant medical history or relevant findings in laboratory exams, electrocardiogram, and physical examination that would have put them at any risk when receiving the study drugs. They had to be willing to follow strict pregnancy prevention measures.

**Fig. 1:**
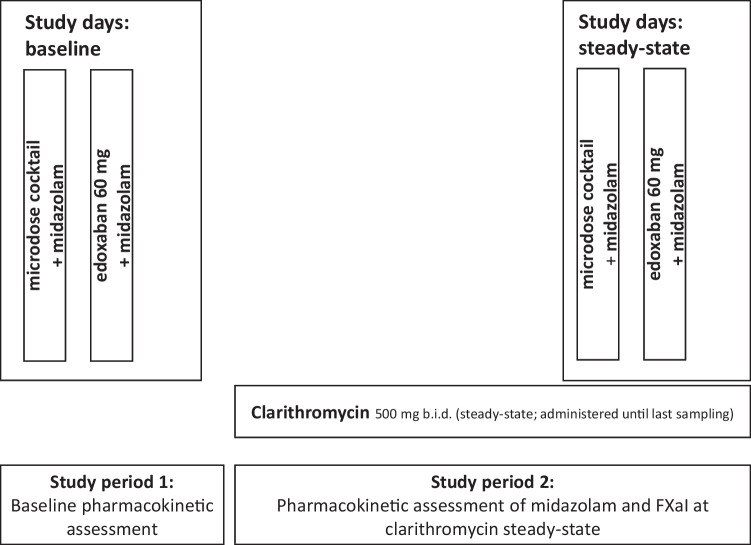
Design of a trial assessing the impact of repeated therapeutic oral doses of clarithromycin on the oral pharmacokinetics of microdosed midazolam, a microdosed factor Xa inhibitor cocktail containing apixaban, edoxaban, and rivaroxaban, and a single therapeutic dose of edoxaban in healthy volunteers. (FXaI factor Xa inhibtors)

The study was an open-label, two-period, one-sequence DDI trial in healthy volunteers to assess the impact of clarithromycin on the pharmacokinetics of 60 mg edoxaban, given as a tablet, and the effect of clarithromycin on apixaban (25 μg), edoxaban (50 μg), and rivaroxaban (25 μg) administered as an oral microdose cocktail [14]. Concurrently, the effect of clarithromycin on CYP3A activity was quantified by means of an oral midazolam microdose (30 μg) (Fig. 1).

CYP3A4 activity was quantified using a microdose of midazolam administered as an oral solution, which was administered directly before administering the FXaI [19, 20]. Clarithromycin was dosed to inhibition steady-state (7-day treatment prior to evaluation of any perpetrator effects and treatment continued until last blood sampling) and administered at a dose of 500 mg b.i.d. to ensure reliable inhibition of hepatic CYP3A4 (mechanism-based inhibition), while maximum CYP3A4 inhibition in the gut wall was expected to occur earlier [21]. Single-dose pharmacokinetics of the FXaI were assessed at baseline and on day 8 (microdosed FXaI cocktail) and day 9 (edoxaban 60 mg) of clarithromycin treatment.

### Quantification of Factor Xa Inhibitors and Midazolam

Venous plasma samples for pharmacokinetic FXaI analyses were collected before and 0.25, 0.5, 0.75, 1, 1.5, 2, 2.5, 3, 3.5, 4, 5, 6, 8, 10, 12, 24 (last sampling after microdoses), and 48 h (last sampling for edoxaban 60 mg) after drug administration. Midazolam pharmacokinetics were assessed using a limited sampling strategy with sampling at 2, 2.5, 3, and 4 h post dose [19, 20]. All samples were processed within 20 min, and plasma was stored at ≤ – 20 °C until analysis. Coagulation effects (international normalized ratio (INR) and activated partial thromboplastin time (aPTT)) were assessed at expected FXaI peak plasma concentrations (3 h post dose) in the accredited central laboratory of the hospital.

Apixaban, edoxaban, midazolam, and rivaroxaban plasma concentrations were quantified using validated highly sensitive ultra-performance liquid chromatography–tandem mass spectrometry (UPLC-MS/MS) methods with accuracy and precision values of less than or equal to ± 15 % [22, 23]. The corresponding lower limits of quantification were 1 pg/ml for microdosed midazolam, 2.5 pg/ml for all microdosed FXaI, and 1 ng/ml for regular doses of edoxaban.

### Pharmacokinetic and Statistical Analysis

Non-compartmental analyses of the FXaI and midazolam pharmacokinetics were performed using Phoenix WinNonlin 8.3 (Certara, Inc., Princeton, NJ, USA). Linear modelling of AUC ratios was done using the R software environment in version 4.0.4 (R Foundation for Statistical Computing, Vienna, Austria). Maximum plasma concentration (C_max_) and the time to reach C_max_ (T_max_) were directly obtained from the pharmacokinetic data. The area under the concentration-time curve from 0 to infinity (AUC_0-∞_) was determined using the log-linear trapezoidal rule and by adding the extrapolated part until infinity. Half-life (t_1/2_) is calculated as $\frac{\ln (2)}{\uplambda \textrm{z}}$, where the elimination rate constant λ_z_ was estimated using log-linear regression of the elimination phase. The apparent oral clearance (Cl/F) was calculated as $\frac{\ \textrm{dose}}{{\textrm{AUC}}_{0\hbox{--} \infty }}$. CYP3A4 activity was determined using a limited sampling strategy using the midazolam AUC_2-4_ as described earlier [19, 20]. Parameters are displayed as geometric means with 95 % confidence interval (CI). Exposure changes are described by evaluating the geometric mean ratio (paired *t*-test on log-transformed values) of AUC_0-∞_ and C_max_ at baseline and under the influence of steady-state clarithromycin (90 % CI). The AUC_2-4 of_ midazolam was evaluated at multiple time points using a repeated-measures analysis of variance after logarithmic transformation.

To evaluate exposure changes between the edoxaban microdose and regular dose, the following analyses were carried out: The agreement of AUC change ratios between both dose groups was evaluated by a Bland-Altman analysis plotting the difference of intraindividual AUC ratios (edoxaban 60 mg and edoxaban 50 μg) against the intraindividual mean of AUC ratios across each dose group. To evaluate P-gp activity as potential predictor for AUC change under inhibition we evaluated the linear regression of the individual baseline AUC with the AUC change.

Statistical analyses and graphical displays were carried out using Prism 9.0 (GraphPad Software Inc., La Jolla, CA, USA). A *p* value < 0.05 was considered significant.

## Results

After having given their written informed consent, we enrolled 12 healthy volunteers (9 females), aged 20–54 years (median 26) with a mean body mass index of 22.5 kg/m^2^ (± standard deviation (SD) 2.36) in this trial. All participants completed the trial. Baseline characteristics are described in Supplemental Table S2.

### Effect of Clarithromycin on FXaI Pharmacokinetics

**Fig. 2:**
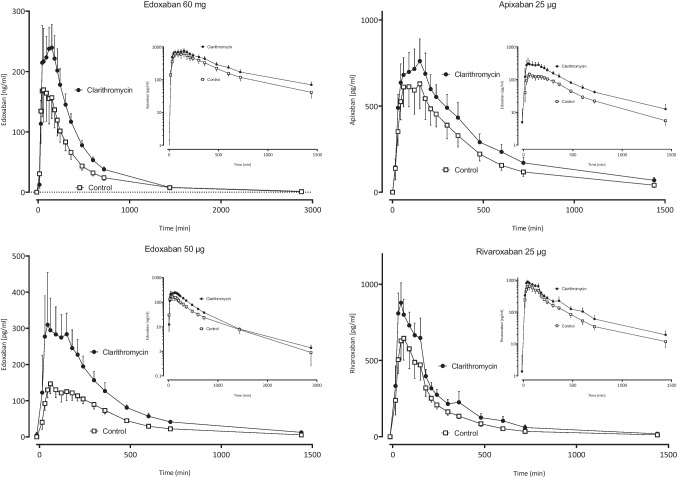
Mean values (± SD) of plasma concentration-time profiles of factor Xa inhibitors (FXaI) after administration of single oral doses before (open squares) and during clarithromycin (solid circles) to 12 healthy volunteers

After administration of the 60 mg dose, the geometric mean ratio (GMR) of edoxaban AUC_0-∞_ increased to 1.53-fold. After microdosed edoxaban with a dose of 50 μg, the dose-normalized absolute exposure increased to 2.03-fold (Fig. 2, Table 1). This increase was larger than the increase observed after the 60 mg dose, which suggests that the microdose moderately overestimates the true extent of the interaction (Table 1). Clarithromycin moderately increased the exposure (Fig. 2, Table 1) and decreased clearance (Table S1) of all FXaI.

**Table 1:** Pharmacokinetic parameters of factor Xa inhibitors at baseline and at clarithromycin steady state

Drug	Pharmacokinetic variable	Baseline		At steady-state clarithromycin		Geometric mean ratio (90 % CI)		*p* value of change
		Geometric mean (95 % CI)		Geometric mean (95 % CI)				
Edoxaban (60 mg)	*AUC*_0-∝_ (min*mg/ml)	70	(60-82)	107	(96–120)	1.53	(1.37–1.70)	< 0.0001
	*C*_max_ (ng/ml)	203	(159–260)	258	(218–304)	1.27	(1.02–1.58)	0.08
μ-Edoxaban (50 μg)	*AUC*_0-∝_ (min*ng/ml)	63	(57–70)	128	(115–143)	2.03	(1.84–2.24)	< 0.0001
	*C*_max_ (pg/ml)	153	(131–180)	341	(280–416)	2.22	(1.92–2.58)	< 0.0001
Apixaban [24]	*AUC*_0-∝_					1.60	(1.51–1.70)	
	*C*_max_					1.30	(1.22–1.38)	
μ-Apixaban (25 μg)	*AUC*_0-∝_ (min*ng/ml)	307	(254–369)	423	(361–496)	1.38	(1.26–1.52)	< 0.0001
	*C*_max_ (pg/ml)	653	(547–780)	764	(652–896)	1.17	(1.15–1.19)	0.02
Rivaroxaban [16]	*AUC*_0-∝_					1.54	(1.44–1.64)	
	*C*_max_					1.40	(1.30–1.52)	
μ-Rivaroxaban (25 μg)	*AUC*_0-∝_ (min*ng/ml)	166	(143–193)	238	(211–270)	1.43	(1.27–1.62)	0.0003
	*C*_max_ (pg/ml)	650	(531–796)	907	(786–1046)	1.40	(1.23–1.59)	0.0008

*AUC*_0-∞_, area under the plasma concentration-time curve extrapolated to infinity (extrapolated fraction < 16 %); CI, confidence interval; *C*_max_, peak plasma concentration; GMR: geometric mean ratio

The interaction data of regular doses of rivaroxaban are extracted from Mueck et al. 2013 [16] and of regular apixaban doses from Garonzik et al. 2019 [24]

**Fig. 3:**
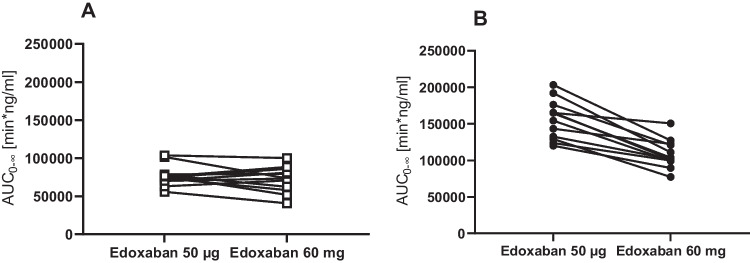
Edoxaban dose-normalized AUCs at baseline (**a**) and during clarithromycin (**b**) after administration of an edoxaban microdose (50 μg) and a high regular dose (60 mg) to 12 healthy volunteers

At baseline, the dose-normalized AUC_0-∞_ of the microdose of edoxaban was similar to the AUC_0-∞_ of the therapeutic dose (GMR 1.08; 90 % CI: 0.97–1.20), while at steady-state of clarithromycin, the GMR of the microdose was significantly larger (1.43; 1.33–1.55) (Fig. 3). There was a significant correlation between the AUC_0-∞_ of edoxaban 60 mg and edoxaban 50 μg both at baseline (Pearson correlation coefficient *r* = 0.57, one-tailed *p* = 0.027) and during clarithromycin steady-state (Pearson correlation coefficient *r* = 0.62, one-tailed *p* = 0.017). In contrast, there was no correlation between the magnitude of AUC_0-∞_ changes after administration of the microdose and the therapeutic dose (data not shown).

There were no baseline characteristics (laboratory values and demographic variables) which correlated to the AUC ratio in either the microdose or the full dose setting (data not shown).

The differences of the AUC_0-∞_ ratios agreed only moderately between the microdose group and the regular dose in a Bland-Altman analysis (Fig. S1). In particular, the pair means of ratios of regular doses and microdose showed an upward shift, suggesting an overestimation of the effect in the microdose setting. AUC changes correlated negatively with the baseline AUC measurements both after a microdose (*p* = 0.010) and a therapeutic dose (*p* = 0.028) (Fig. S2).

### Assessment of CYP3A Activity

Midazolam AUC_2-4_ was 6.4 min*ng/ml (90 % CI 4.9–8.3 min*ng/ml) and 40.3 (35.1–46.2) min*ng/ml at baseline and at clarithromycin steady-state, and the GMR was 6.30 (90 % CI 5.16–7.70), confirming substantial inhibition of CYP3A activity (*p* < 0.0001) (see Table S1). The mean increase of midazolam AUC_2-4_ was significantly different from the mean increase of the AUC_0-∞_ of individual FXaI (*p* < 0.0001). Tested as paired values to evaluate intraindividual correlation of changes in a participant, there was no significant linear correlation between the increase of midazolam AUC_2-4_ and the increase of AUC_0-∞_ of any of the FXaI edoxaban, apixaban, or rivaroxaban.

### Effect on Coagulation Markers

Regular edoxaban doses significantly increased aPTT and INR values, and anticoagulation further increased during clarithromycin (Table 2). There was also a small anticoagulant effect after the microdose cocktail but only during co-administration of clarithromycin.

**Table 2:** Coagulation parameters measured 180 min after administration of a microdosed factor Xa inhibitor cocktail and after a regular 60-mg dose of edoxaban

Parameter	Baseline		Edoxaban 60 mg alone		Edoxaban 60 mg with clarithromycin		Change during clarithromycin		*p* Value
	*Mean*	± *SD*	*Mean*	± *SD*	*Mean*	± *SD*	*GMR*	*(95 % CI)*	
aPTT	24.9	1.0	33.4	2.85	40.2	3.68	1.61	(1.51–1.71)	< 0.0001
INR	1.02	0.06	1.23	0.11	1.43	0.17	1.39	(1.31–1.47)	< 0.0001
Parameter	Baseline		FXaI cocktail alone		FXaI cocktail during clarithromycin		Change during clarithromycin		*p* Value
	*Mean*	± *SD*	*Mean*	± *SD*	*Mean*	± *SD*	*GMR*	*(95% CI)*	
aPTT	24.9	1.0	25.3	1.0	25.9	1.2	1.04	(1.02–1.06)	0.01
INR	1.02	0.06	1.04	0.05	1.05	0.06	1.02	(1.00–1.05)	ns

aPTT, activated partial thromboplastin time; GMR, geometric mean ratio; INR, international normalized ratio; ns, not significant; SD, standard deviation

### Safety

Overall, 27 adverse events (AE) occurred in 11 of 12 participants, all of which were deemed possibly related to the trial. All AE were transient, none was serious, and none resulted in a withdrawal from the trial. The most frequent AE was dysgeusia reported by 9 participants while taking clarithromycin. Other AE occurring more than once were abdominal pain (*n* = 4), headache (*n* = 3), and diarrhea (*n* = 2), which all occurred under clarithromycin treatment.

## Discussion

### Influence of Clarithromycin on the Pharmacokinetics of 60 mg Edoxaban

In this first trial, examining the potential DDI of the P-gp and CYP3A4 inhibitor clarithromycin, clarithromycin moderately increased edoxaban exposure by 53 %. This is similar to the magnitude of drug interactions observed with therapeutic doses of apixaban (59 % [24]) and rivaroxaban (54 %, [16]). Overall, these exposure changes are less than those observed when therapeutic doses of FXaI were combined with the strong P-gp inhibitor ketoconazole (edoxaban, + 87 % [25]; apixaban, + 99 % [26]; rivaroxaban, + 158 % [16]). In addition to its higher potency as CYP3A inhibitor, ketoconazole also inhibits organic anion transporter 3, breast cancer resistance protein, CYP3A, and CYP2J2 [27, 28]. Because CYP2J2 appears to be the dominant hydroxylating isozyme in the metabolism of rivaroxaban *in vitro* [29], and CYP3A is presumably the dominant pathway of apixaban [30], it must be expected that there are differences for the effects on DDI of the perpetrator drugs ketoconazole and clarithromycin for apixaban and rivaroxaban.

Both efficacy and safety of FXaI treatment appear to be linked to FXaI exposure [31], and retrospective evidence from large cohort analyses mostly, albeit not always [32], suggests that co-medication increasing FXaI exposure is linked to major bleeding events [21, 33, 34], while combinations decreasing exposure increase the risk of thromboembolic events by approximately 60 % and more than double the risk of strokes [21, 35]. Epidemiological evidence suggests that the approximately 40 % exposure increase of apixaban and rivaroxaban caused by verapamil or diltiazem does not increase bleeding risk [36]. In contrast, in epidemiological studies evaluating patients mainly anticoagulated with FXaI, the risk of hospital admission due to major bleeding events was increased by 71 % during clarithromycin compared to azithromycin [18]. However, in this latter study, FXaI concentrations were not measured, and a significant proportion of patients had renal impairment, a comorbidity, which may have potentiated the impact of CYP3A inhibition [37]. To date, no clear therapeutic range has been defined, and it is not known what magnitude of exposure change is clinically relevant. Generally, information in the drug label approved by the authorities indicates that no dose adjustment is needed as long as exposure increases are ≤ 90 % (edoxaban [11]) or ≤ 100 % (apixaban [12], rivaroxaban [14]) but a clear threshold has not been defined.

### Influence of Clarithromycin on the Pharmacokinetics of Microdoses of Edoxaban, Apixaban, and Rivaroxaban

Exposure increases observed with microdoses of apixaban and rivaroxaban agreed well with the magnitude of interaction reported with regular doses [11, 16]. In contrast, after administration of a microdose of edoxaban, the increase of exposure was significantly, albeit only moderately larger, slightly overestimating the absolute increase of exposure by the interaction of a regular high dose. The reason for these findings is not clear yet: The fact that dose-normalized AUC_0-∞_ values of the microdose and regular dose did not differ at baseline excludes pharmacokinetic nonlinearity in the absence of a perpetrator and formulation differences (solution of the microdose vs. tablet) as a possible cause. In addition, edoxaban does not have any perpetrator properties that could explain a potential difference of the effect of a therapeutic dose and a microdose. Therefore, the difference is unlikely due to a decrease in clarithromycin exposure under the influence of a single therapeutic dose of edoxaban. Moreover, we assume that clarithromycin exposure and thus its inhibitor effects were similar in the two phases as shown by similarly decreased CYP3A activity. We have recently shown that this FXaI microdose cocktail can predict the magnitude of the known ketoconazole-induced exposure changes observed with regular doses of FXaI [14]. This study confirms these already published findings for rivaroxaban, which was found to have an increased exposure of approximately 50–100 % with clarithromycin [11, 16] and also the corresponding findings of a 60 % increase for apixaban [15].

Clarithromycin is a macrolide inhibiting multiple relevant metabolic pathways including CYP3A [38], P-gp [39], and organic anion-transporting polypeptides (OATP) 1B1 and 1B3 [40]. Therefore, changes in exposure from drug interactions by clarithromycin may result from changes in either of the affected pathways. As a consequence of the very differing shares of metabolic pathways for the different FXaI [5], inhibition of individual and multiple pathways will have variable impact on exposure changes of the victim drugs, which agrees well with the fact that CYP3A is known to contribute mainly to apixaban [30, 41], less to rivaroxaban metabolism [16], whereas carboxylesterase 1 and P-gp are the dominant clearance pathways of edoxaban [42] with a contribution of CYP3A to the overall metabolic clearance of only 1 % [43]. However, despite this fact, the overall extent of interaction for edoxaban with clarithromycin was similar to the interaction observed with the other FXaI. This suggests that in the case of edoxaban, the interaction with clarithromycin is largely caused by modulation of P-gp. In theory, inhibition of carboxylesterase 1 could also cause such a phenomenon, but clarithromycin has not been reported to have such a property, and it is not converted to acyl glucuronides [44], which have been identified as carboxylesterase inhibitors [45].

### Differences in the Pharmacokinetics of Microdoses and Therapeutic Doses of Edoxaban

To further evaluate the usefulness of microdose cocktail approaches, the relative divergence of the measured changes in plasma levels between microdose and 60-mg dose of edoxaban during clarithromycin was addressed. Edoxaban is actively secreted via P-gp both into urine and feces [46]. P-gp inhibition can reduce intestinal excretion during the absorption phase and biliary and renal tubular secretion during elimination, thus resulting in increased exposure. When comparing the effect of oral clarithromycin on the paradigm P-gp marker substrate digoxin given intravenously and orally, the largest perpetrator effect occurred in the absorption process in the gut with only minor reductions (15 %) of renal tubular secretion [47, 48]. While our study could not disentangle the specific mode of interaction with edoxaban, it reveals that the microdose of edoxaban is more affected by the macrolide than a regular dose, leading to overestimation of the true extent of interaction in the clinical setting when regular edoxaban doses are used. These suggest that full inhibition of pathways contributing to Cl/F by clarithromycin may be more relevant for a microdose than for a therapeutic dose, which may use alternative uptake pathways. Edoxaban is mainly transported by P-gp, CYP3A does not contribute to its clearance and F to any relevant extent [43], and selective inhibition of CYP3A (by voriconazole) does not affect its pharmacokinetics [49].

There is a significant intraindividual correlation between the AUC_0-∝_ of the microdose and the therapeutic dose of edoxaban. Interindividual differences of the expression and activity of P-gp (and/or similar transporters) are likely to influence edoxaban AUC_0-∝_ differently depending on its dose. Consistent with this, the AUC_0-∝_ of edoxaban shows evidence of less variability and correlation between microdose and therapeutic dose when clarithromycin is administered. This is most likely due to the elimination of variations in the activity of basic enzymes and transporters, e.g., of P-gp. The shift to overestimation of exposure has additionally been visualized via Bland-Altman analysis (Fig. S1).

These findings are further supported by the fact that the baseline AUC_0-∝_ of edoxaban and the factor of AUC_0-∝_ increase during treatment with clarithromycin were significantly correlated. Therefore, individuals with lower baseline exposure had a larger increase of AUC_0-∝_, which might indicate that their baseline P-gp activity was higher and that these differences in the extent of interaction are likely transporter-mediated.

### Limitations

The observed inverse relationship between edoxaban exposure at baseline and extent of the interaction with clarithromycin could be the result of interindividual differences in P-gp activity or clarithromycin exposure; neither of them has been studied. Furthermore, variations in exposure could also be caused by different release characteristics of the edoxaban tablet compared to its microdose solution. We did not genotype our participants and did not power the trial for genetic differences because, in an earlier trial, P-gp haplotypes did not predict the extent of clarithromycin-induced exposure changes of rivaroxaban [11, 16]. Because clarithromycin is a mechanism-based inhibitor, which was administered until an inhibition steady-state was reached, and because CYP3A inhibition was closely followed using midazolam, it is very unlikely that small fluctuations in clarithromycin plasma concentration would have affected edoxaban pharmacokinetics.

## Conclusion

This paper has shown that clarithromycin increases exposure of a therapeutic dose of edoxaban in healthy volunteers 1.53-fold, which is not considered clinically relevant. However, FXaI are often prescribed to patients with impaired renal function, which may further increase the exposure of FXaI when co-administered with clarithromycin [37]. Therefore, appropriate dose reduction for edoxaban may be needed for patients with renal impairment when clarithromycin is concomitantly used. Furthermore, our trial suggests that edoxaban microdoses might overestimate the extent of interaction with P-gp inhibitors.

### Supplementary information

ESM 1 (PDF 231 KB)

## Data Availability

Data will be made available by the corresponding author of this publication upon personal request.
