# Supplementary material for: Effect of Clarithromycin, a Strong CYP3A and P-glycoprotein Inhibitor, on the Pharmacokinetics of Edoxaban in Healthy Volunteers and the Evaluation of the Drug Interaction with Other Oral Factor Xa Inhibitors by a Microdose Cocktail Approach
Source: Cardiovasc Drugs Ther. 2023 Mar 4;38(4):747–56. doi: 10.1007/s10557-023-07443-2 (PMC11266212; doi:10.1007/s10557-023-07443-2)
Supplement: Supplementary file 1 — ESM 1 (PDF 231 KB) [file 10557_2023_7443_MOESM1_ESM.pdf]

**Supplemental Material**

# Effect of clarithromycin, a strong CYP3A and P-glycoprotein inhibitor, on the pharmacokinetics of edoxaban in healthy volunteers and the evaluation of the drug interaction with oral factor Xa inhibitors by a microdose cocktail approach

---


**Alexander Lenard[1]**

**Simon A. Hermann[1]**

**Felicitas Stoll[1]**

**Juergen Burhenne[1]**

**Kathrin I. Foerster[1]**

**Gerd Mikus[1]**

**Andreas D. Meid[1]**

**Walter E. Haefeli[1]**

**Antje Blank[1;#]**

[1] Department of Clinical Pharmacology and Pharmacoepidemiology, University of Heidelberg, 69120 Heidelberg, Germany.

Supplemental Table S1: Pharmacokinetic parameters of factor Xa inhibitors and midazolam as marker for CYP3A activity

| **Drug (dose)** | **Pharmacokinetic variable** | | **Baseline** | | **At clarithromycin steady-state** | | **p-value of change** |
|---|---|---|---|---|---|---|---|
| | | | *GM* | *(95 % CI)* | *GM* | *(95 % CI)* | |
| Edoxaban (60 mg) | Cl / F | [L/h] | 51 | (44 – 60) | 34 | (30 – 38) | < 0.0001 |
| | $t_{1/2}$ | [min] | 7.3 | 6.2-8.6 | 7.2 | 6.4-8.0 | 0.8 |
| | $T_{max}$ | [min] | 76 | 46-95 | 100 | 59-125 | 0.3 |
| | Vz | [L] | 594 | 412-711 | 361 | 288-419 | 0.0006 |
| µ-edoxaban (50 µg) | Cl / F | [L/h] | 48 | 43-53 | 23 | 21-26 | < 0.0001 |
| | $t_{1/2}$ | [min] | 5.6 | 4.9-6.5 | 6.3 | 5.7-7.1 | 0.3 |
| | $T_{max}$ | [min] | 70 | 45 - 85 | 103 | 44 - 132 | 0.4 |
| | Vz | [L] | 399 | 327-459 | 217 | 191-240 | 0.0002 |
| µ-apixaban (25 µg) | Cl / F | [L/h] | 4.9 | 4.1-5.9 | 3.5 | 3.0-4.2 | < 0.0001 |
| | $t_{1/2}$ | [min] | 7.1 | 6.4-7.9 | 8.1 | 7.4-8.9 | 0.14 |
| | $T_{max}$ | [min] | 114 | 74-141 | 134 | 96-164 | 0.3 |
| | Vz | [L] | 52 | 43-59 | 43 | 35-50 | 0.06 |
| µ-rivaroxaban (25 µg) | Cl / F | [L/h] | 9.0 | 7.8-11.0 | 6.3 | 5.6-7.1 | 0.0003 |
| | $t_{1/2}$ | [min] | 6.3 | 5.3-7.5 | 5.7 | 5.2-6.3 | 0.5 |
| | $T_{max}$ | [min] | 55 | 47-62 | 55 | 38-63 | 0.5 |
| | Vz | [L] | 85 | 67-100 | 53 | 45-60 | 0.0042 |
| µ-midazolam | $AUC_{2-4}$ | [min*ng/mL] | 6.4 | 5.1-7.9 | 40.3 | 35.9-45.1 | < 0.0001 |

*GM: geometric mean*

Supplemental Table S2: Baseline characteristics of the 12 study participants

| **Laboratory parameter** | | **Mean** | **Standard deviation** | **Normal range** |
|---|---|---|---|---|
| Albumin | [g/L] | 46.5 | 4.29 | 30 - 50 |
| Alanine aminotransferase | [U/L] | 17.67 | 11.9. | < 50 |
| Alkaline phosphatase | [U/L] | 56.58 | 16.3 | 40 - 130 |
| Aspartate aminotransferase | [U/L] | 21.58 | 5.42. | - < 46 |
| γ-glutamyltranspeptidase | [U/L] | 12.08 | 8.08 | < 50 |
| Bilirubin | [mg/dL] | 0.675 | 0.32 | < 0.3 |
| Calcium | [mmol/L] | 2.35 | 0.15 | 2,11 - 2,59 |
| Chloride | [mmol/L] | 104.8 | 1.27 | 98 - 111 |
| Potassium | [mmol/L] | 3.80 | 0.23 | 3,4 - 5,0 |
| Sodium | [mmol/L] | 140 | 1.66 | 135 - 146 |
| Phosphate | [mmol/L] | 1.05 | 0.17. | 0,84 - 1,45 |
| Total protein | [g/L] | 70.4 | 3.86 | 60 - 80 |
| Glucose | [mg/dL] | 82.1 | 9.10 | 65 - 110 |
| Urea | [mg/dL] | 23.0 | 6.41 | < 45 |
| Creatine kinase | [U/L] | 147 | 165 | < 190 |
| Creatinine | [mg/dL] | 0.70 | 0.12 | 0,6 - 1,4 |
| Estimated creatinine clearance | [mL/min] | 129 | 28.9 | > 60 |
| Lipase | [U/L] | 46.0 | 13.1 | 19 - 63 |
| P-amylase | [U/L] | 30.8 | 13.3 | 8 - 53 |
| Erythrocytes | [/pL] | 4.76 | 0.37 | 4,3 - 6,1 |
| Hemoglobin | [g/dL] | 13.6 | 1.23 | 13 – 17 |
| Hematocrit | [L/L] | 0.40 | 0.03 | 0,38 - 0,52 |
| Leucocytes | [/nL] | 7.06 | 1.96 | 4 - 10 |
| Lymphocytes | [/nL] | 1.70 | 0.40 | 1,0 - 4,8 |
| Monocytes | [/nL] | 0.40 | 0.10 | 0,2 - 0,8 |
| Neutrophils | [/nL] | 4.78 | 1.64 | 1,8 - 7,7 |
| Basophils | [/nL] | 0.03 | 0.01 | < 0,2 |
| Eosinophils | [/nL] | 0.10 | 0.07 | < 0,5 |
| Platelets | [/nL] | 266 | 45.4 | 150 - 440 |
| Activated partial thromboplastin time | [s] | 24.63 | 1.20 | < 35 |
| International normal ratio | | 1.02 | 0.05 | < 1.2 |

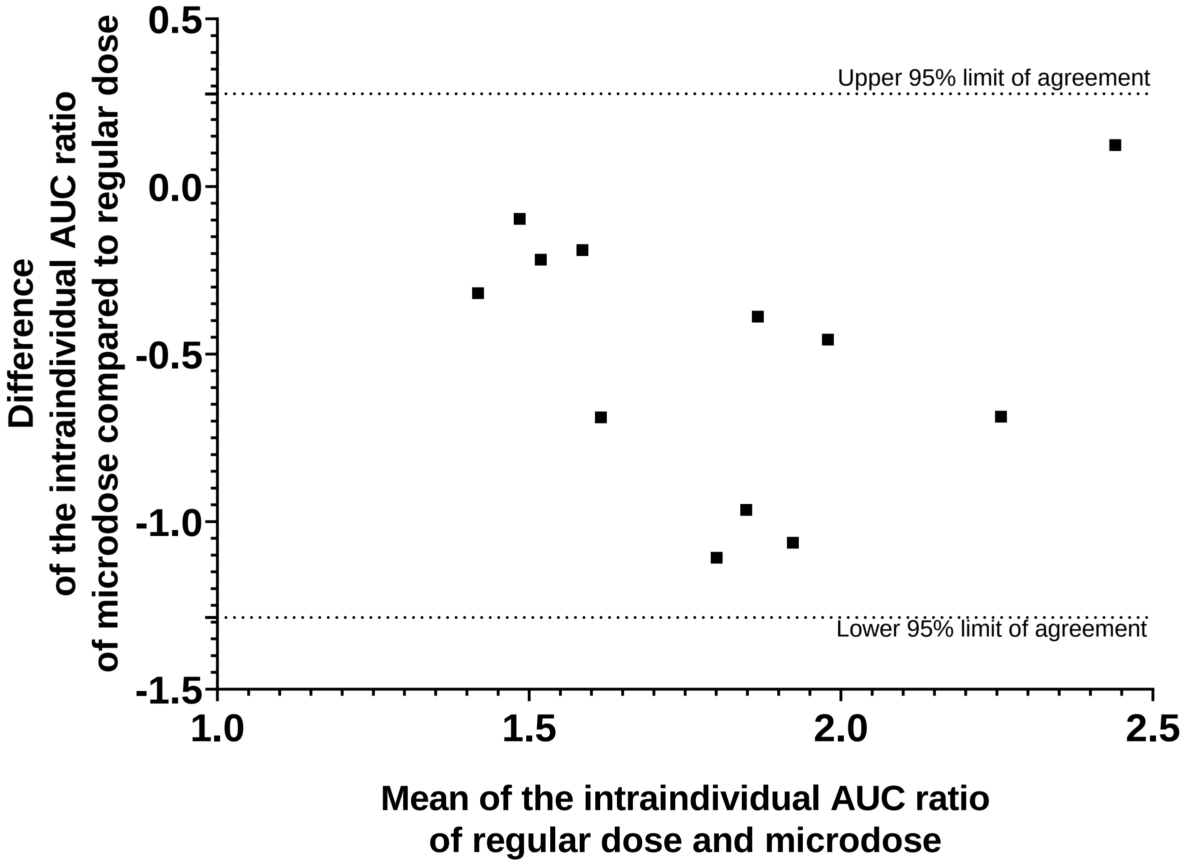


**Supplemental Figure S1** Bland-Altman plot for agreement between regular dose and microdose of edoxaban. The mean of intraindividual AUC ratios under clarithromycin (per volunteer/mean of AUC of edoxaban 60 mg and AUC of edoxaban 50 µg) were plotted against the difference of the intraindividual AUC ratio of the microdose compared to the regular dose.

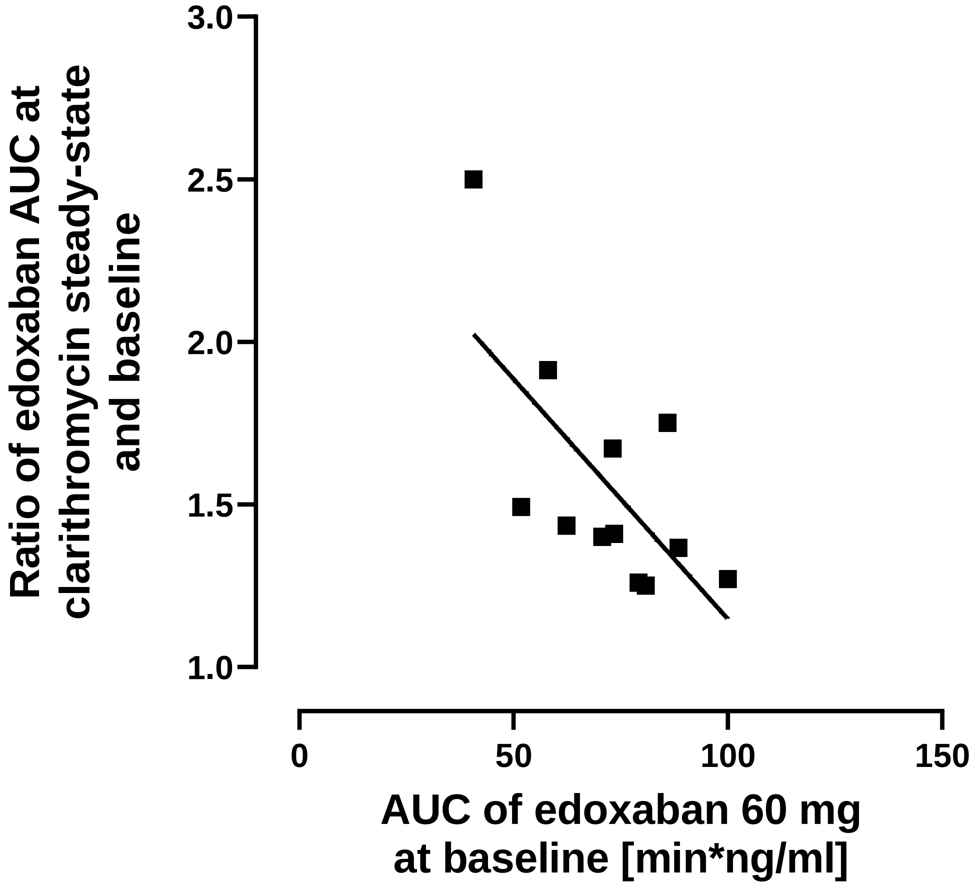


**Supplemental Figure S2** Regression of the individual baseline exposure of edoxaban 60 mg ($r^2$ = 0.47. p = 0.03). as a marker mainly influenced by P-glycoprotein activity with the ratio of the AUC increase at clarithromycin steady-state.

.
